# Kantian political philosophy, coercion, and public health

**DOI:** 10.1007/s40592-025-00239-0

**Published:** 2025-04-07

**Authors:** Remco J. L. van Dijk, Justin S. Bernstein

**Affiliations:** 1 Vrije Universiteit Amsterdam, Amsterdam, Netherlands; 2 Regional Public Health Service (GGD) Gelderland Zuid, Nijmegen, Netherlands

**Keywords:** Kantian political philosophy, Public health, Coercion, Freedom, Vaccination

## Abstract

Many public health policies are coercive and therefore, they require moral justification. Kantian political philosophy is an under-explored but appealing approach to public health ethics. According to the Kantian approach, which is centred around freedom as independence, the state has an important role in protecting that freedom. In doing so, the state is justified to use coercion. To illustrate the Kantian approach, we consider its implications in the context of coercive vaccination policy. We show coercive vaccination policies are justified, because the state is needed to provide determinacy, because such policies are needed to guarantee the systematic enjoyment of the right to freedom, and because such policies reduce the risk for dependence on others.

## Kantian political philosophy, coercion, and public health

Many public health policies threaten sanctions, including the use of force, if the public does not comply. To name just a few examples, if motorcyclists refuse to wear helmets despite the existence of motorcycle helmet mandates, individuals refuse to pay sugary beverage taxes, or they illegally purchase cigarettes in the face of a cigarette ban, they face a variety of legal penalties. Similarly, if vendors refuse to observe sanitary standards, fail to include nutritional information on their products despite requirements to do so, or insist on selling foods with trans-fats in the wake of a trans-fat ban, they also face legal penalties. Such policies give rise to a fundamental question in public health ethics, a question of political legitimacy^1^: what, if anything, permits governments to issue and enforce legal norms that aim to promote the health of a population? Our goal in this paper is to articulate one approach to answering this question that has not been considered at any meaningful length in the public health ethics literature: a broadly Kantian *political* approach to public health.^2^ We illustrate this approach by considering the implications of a Kantian political approach to vaccination policies. We do so for three reasons.

First, despite the influence of Kantian ethics in biomedical ethics—especially the principlist articulation of the principle of autonomy and its accompanying emphasis on not using patients or research subjects as a mere means^3^—there is far less discussion of Kantian *political* philosophy in the public health ethics literature. Indeed, in many instances where Kantian normative philosophy does get discussed in the public health context, authors apply familiar moral principles that Kantians consider in the interpersonal context without attending to Kantian work on political morality. For example, many have considered whether various public health policies would be objectionable because they involve treating people like a mere means^4^—despite the fact that Kant and Kantians do not treat this principle, as applied to the interpersonal ethical context, to be the appropriate basis for assessing the moral justifiability of public policies. Accordingly, Kantian political philosophy is a strikingly under-explored approach to public health ethics generally.

Second, the Kantian view of coercion and freedom differs from most discussion in the public health ethics literature. To the extent that the Kantian approach is promising or plausible, generally, it constitutes an important rival to other approaches. Such rival approaches include a broadly “Millian” understanding of political morality,^5^ where “the harm principle” takes centre stage; the influential “Nuffield Ladder”,^6^ which treats different interventions as requiring greater justification the more liberty-restricting they are; as well as approaches to public health ethics that openly eschew treating freedom as centrally important—such as broadly utilitarian approaches,^7^ feminist approaches,^8^ or views that treat concepts like solidarity as central.^9^ The Kantian approach differs importantly from these views, for reasons we will go on to discuss—and, we will contend, it enjoys at least some theoretical advantages over these rivals.

Third, we consider vaccination policy because we believe it can help to illustrate the promise and appeal of the Kantian view—even in a case where a concern for coercion and respecting the value of freedom would appear to militate against the policy. On its face, attaching disincentives to foregoing a (safe and effective) medical intervention would seem to be a paradigmatic example of the kind of policy that Kantian *moral* philosophy would oppose. But, as we will suggest, if one adopts Kantian *political* philosophy as an approach to public health ethics, this affords the resources to both provide a nuanced diagnosis of the moral issues at stake with such policies and make plausible arguments for justifying such policies. Moreover, the Kantian view justifies such policies by appealing to how respecting the value of freedom can permit, or even require, state coercion. For reasons we will discuss below, this framing seems promising because it differs from existing defences of vaccine mandates that invoke values other than freedom.

The structure of the paper is as follows. First, we summarise the key features of Kant’s political philosophy—his understanding of freedom and coercion and his account of the role of the state in protecting freedom and the appropriate uses of state coercion (Sect. 1). In Sect. 2, we spell out the implications of a Kantian approach to justifying vaccine mandates. In Sect. 3, we highlight some theoretically appealing features of the Kantian approach to thinking about coercion and freedom in public health ethics, more generally.

Before beginning, we should make explicit a methodological assumption. Namely, we are interested in a Kant*ian* account of coercion; we are not engaged in Kant scholarship. Accordingly, we draw on contemporary Kantians—especially the influential work of Arthur Ripstein—who have played a significant role in articulating and defending Kantian political philosophy as a plausible rival to other contemporary views in political philosophy. If one objects to characterising such work as Kantian because they disagree on various points of Kant scholarship, then they may use some other label for the view; the promise of the view does not, in our view, turn on whether it constitutes the best interpretation of Kant’s political philosophy.

## Kantian political philosophy: freedom, coercion, and the state

### Freedom

The core idea in Kant’s political philosophy is that every rational being has a right to be free. According to Kant, this right to freedom is the only original right humans have—he calls it the innate right of humanity.^10^ The innate right of humanity needs no affirmative act to establish it, and all further rights people may have are based on this innate right to freedom (Ripstein 2009, pp. 31–35).

This innate right to freedom, as can be seen in Kant’s definition, must be understood in the sense of freedom as *independence* (Ripstein 2009, p. 17; Rostbøll 2016; Unberath 2008, p. 324).^11^ Being free in the sense of independence means that every person is their own master, and thus also that no other person is their master. One person must not be subject to or be constrained by the choice of another (Kleingeld 2020, pp. 110–111; Ripstein 2009, pp. 30–31).^12^ For each to be free, everyone must be able to set their own ends and pursue those ends with the means available to them.^13^

If that ability to set and pursue your own ends is taken away by others, your freedom as independence would be infringed. Enjoying freedom as independence, in turn, marks the distinction between persons and things.^14^ On the Kantian view, then, interfering with someone’s freedom as independence involves treating an agent as a thing rather than a rational agent, and such treatment is morally impermissible.^15^ It follows that freedom also acts as a constraint on the conduct of others: others cannot restrict my freedom by making me subject to their choice. So, each person is entitled to use their powers as they see fit if that is consistent with the ability of others to do the same with theirs (Ripstein 2009, pp. 36–42).

Putting these pieces together, we arrive at Kant’s Universal Principle of Right: “Any action is *right* if it can coexist with everyone’s freedom in accordance with a universal law, or if on its maxim the freedom of choice of each can coexist with everyone’s freedom in accordance with a universal law.” (Kant 1797/1996b, 6:230, emphasis in original).^16^ In a situation where free individuals coexist and no one makes other persons subject to their own purposes—in other words, when everyone acts rightfully, in accordance with the Universal Principle of Right—separate or cooperative exercises of individual powers will form a system of equal freedom and equal independence (Ripstein 2009, p. 39; Rostbøll 2016, p. 795). In such a system, everyone’s right to freedom as independence is respected, and as such, no one is treated as a mere thing, and everyone is respected as a moral equal.

### Coercion

This analysis of freedom sets the stage for the Kantian analysis of coercion: coercion is the interference with or the restriction of freedom. There are two ways in which my freedom can be interfered with. First, my powers can be usurped by others. This means another person exercises my powers for their own purposes or gets me to exercise my powers for their own purposes. The other makes me do something I would not have done otherwise, and by doing that, they use me and violate my entitlement to be my own master. Familiar examples of this first type of interference are threats to use force, fraud, or manipulation. Second, my powers can be destroyed or injured. That means another person reduces my ability to set and pursue my own purposes, thereby making themself my master because the other decides that I can no longer use my powers. Examples of this second type of interference are injuring one’s person or one’s property, and constraining them physically (Ripstein 2004, p. 21; Ripstein 2009, pp. 43–45).

Coercion, understood in this way as the restriction of freedom, is prima facie objectionable and unrightful because it is inconsistent with the system of equal freedom required by the Universal Principle of Right: if one person restricts the freedom of another person, there is no longer equal freedom for all. For example, a kidnapper kidnapping a victim is using unrightful coercion—violating the Universal Principle of Right—because it restricts the freedom of the victim, making the victim subject to the choice of the kidnapper.

However, coercion can also take a legitimate and rightful form, when someone restricts a restriction of freedom. For example, in the kidnapping case, the victim could use force to escape from the kidnapper (or someone else could use force to rescue the victim). This would be a use of coercion directed at the kidnapper, making the kidnapper subject to the choice of the victim (or the other person rescuing the victim), but it would be a rightful and legitimate use of coercion, because it is a so-called “hindrance to a hindrance to freedom”: it is a restoration of the original right to freedom as independence (González 2010; Ripstein 2008). For the Kantian, every individual has an entitlement to coerce others when their right to freedom as independence is violated by those others, just like in the kidnapper case (Ripstein 2009, pp. 54–56). In this light, the Kantian right to freedom, as in the right to independence from the choice of others under universal law, can also be framed as the right or the entitlement to constrain the conduct of others, when that constraint forms a hindrance of a hindrance to freedom (Hodgson 2010; Ripstein 2009, pp. 217–218; Valentini 2012, p. 454).

### The state

According to Kantians, the state provides the only possible way in which a plurality of individuals can interact in a way that respects the freedom of each. The state does so by ensuring, in light of the Universal Principle of Right, that everyone enjoys equal freedom, both in enjoying the innate right of freedom as independence, as well as in enjoying private rights and entitlements that follow from the innate right of freedom.^17^ In other words, the state is needed to create a rightful condition in which each person can be independent.

The state is needed for this, because only the state can make laws from the ‘omnilateral standpoint’: laws that make sure citizens have the freedom to make choices, reconciling that freedom with the freedom of those others who are bound by those choices (Ripstein 2009, pp. 148–159). Kantians claim the omnilateral will is the only will that is ‘lawgiving’, because a unilateral will (the will of, for example, an individual, or a private entity) cannot make laws and impose obligations on others without violating the Universal Principle of Right. The idea of the Kantian political system is that the state is acting on behalf of the citizens as a collective body.^18^

The Kantian state, with its norms and institutions, provides three requirements that ensure the rightful condition: authorisation, assurance, and determinacy (Ripstein 2009; Chap. 6).^19^ If those requirements are not met, people will find themselves in a state of nature, where “arbitrary individual force prevails” (Ripstein 2004, p. 3), and where rights are not conclusive but only provisional (Stone and Hasan 2022).^20^ In providing this rightful condition of equal freedom, the state has an integral role to hinder hindrances to freedom. The state is justified, according to Kantians, to use coercion insofar as the use of coercion is necessary to hinder hindrances to freedom, thereby securing the rights of citizens (Ripstein 2009, pp. 176–181). The basic justificatory rationale for the state’s public coercive authority is thus its capacity to secure the individual right to freedom as independence.

The Kantian state can only make arrangements and laws that guarantee the systemic enjoyment of the right to freedom. Such a law might include some specific restrictions of freedom, only in order to guarantee the systemic preconditions for freedom as independence (Ripstein 2009, p. 206).^21^ Individuals can be compelled to cooperate when preconditions of sustaining a condition of equal freedom as independence are at stake. They can thus be compelled to contribute to social projects if those projects guarantee or sustain the rightful condition of equal freedom (Ripstein 2009, p. 256). The government can compel people to contribute to such public goods, without relying on the benefits or burdens those individual contributions will actually bring (Ripstein 2022, p. 25).^22^

## Kantian justifications for coercive vaccination policy

We have just provided a brief overview of Kantian political philosophy, its understanding of freedom and coercion, and the role it accords to the state. In what follows, we would like to illustrate what it would look like to apply this framework to thinking about the justification of coercion in public health policy. To do so, we will consider a policy that has received a great deal of attention in recent years: vaccine mandates.

On the Kantian account, coercive vaccination policy aimed at increasing vaccination rates can be justified. We use “coercive vaccination policy” broadly, and the arguments we give are abstract enough to be compatible with several different kinds of policies. But we will focus on a paradigmatic kind of policy that gets discussed to increase vaccination rates: vaccine mandates. Such mandates restrict access to goods and services for those who refuse vaccination for themselves or their children.^23^ We assume these policies count as coercive on the Kantian account because such mandates usurp the powers of others—they issue threats to get people to behave in ways they otherwise would not. Yet despite being coercive, we present four arguments for the claim that such coercion is rightful, and thus that such policies are justified.

In advancing these arguments, we make some assumptions to make the discussion clearer. We will assume there is an effective vaccine available which protects from infection with a dangerous infectious disease, and when enough people are vaccinated, herd immunity will emerge, which is especially relevant for vulnerable people.^24^ Diseases we have in mind that could be suitable targets for coercive vaccination policies are, for example, measles and polio. Furthermore, we assume that the proposed coercive vaccination policy is effective in reducing vaccine refusal. Modifying any of these assumptions would lead to further interesting discussion, but we lack room to explore these more complicated scenarios. Making the Kantian case for vaccine mandates in the relevant idealised circumstances is both interesting enough in its own right, and it suffices to highlight the potential of Kantian political philosophy in public health ethics.

### Protecting the right to public use

The “protecting the right to public use” argument proceeds as follows. Each individual has an equal right to enter into safe public spaces. Doing so is necessary for individuals to enjoy their freedom as independence. In the absence of sufficient vaccination, however, vulnerable individuals cannot safely use public spaces. The state has a proper role to ensure that each enjoys the right to safely use such public spaces. In some circumstances, enacting coercive vaccination policy is the best way to ensure that public spaces are safe for vulnerable people to use. So, the state acts within its proper role in the relevant circumstances when it enacts such policies.

In our view, the premises in this argument that most require elaboration are (1) individuals have the right to use public spaces and (2) it’s the proper role of the state to ensure that each enjoys this right.

With regard to (1), there are two related supporting arguments for the idea that individuals have the relevant right to use public spaces. The first involves an analogy with privately owned property. Kant argues that certain ends can only be accomplished if individuals can come to privately own property. If I can count on my exclusive use of some goods only if they are currently in my hands, then this will severely limit my ability to enact longer-term plans that require setting those goods down. Precluding private ownership of property, then, would limit human freedom for no reason.^25^ Accordingly, Kant argues that it must be possible for people to come to own private property.

A similar thought applies to public spaces. Being unable to rightfully use public spaces—spaces to which all individuals have a right to use—severely limits each individuals’ ability to use their own means to pursue their ends. If I am free to use my property and my body as I see fit but only within the confines of my own home, there’s a sense in which my freedom to set and pursue ends is severely constrained. Moreover, lacking the right to use public spaces would appear to limit human freedom for no reason. So there need to be some public spaces that allow each to set and pursue their purposes.

This leads us to (2)—the claim that it’s the proper role of the state to establish and enforce the relevant rights. Just as a state is necessary for establishing and enforcing private property rights for the Kantian, a state must also fulfil this function for public spaces. As noted, the state is needed to determine boundaries in cases where there is indeterminacy when it comes to private property rights.^26^ A rightful state is similarly needed to resolve disputes about the extent and limits of public spaces. For example, my decision to park a car on the side of a public road will sometimes be acceptable. Parking in the middle of a public road, by contrast, precludes others from accessing and using the public space. But it’s not as though there’s some sort of pre-institutional fact of the matter about the exact contours of one’s rights to park one’s car and the claims of others against you parking your car somewhere. A legitimate state, then, can both determine what constitutes blocking a public road, and it can enjoy recognition as the authority to do so.^27^

There are two especially salient ways in which states can use coercion to ensure individuals do their part in ensuring the existence and use of public space. First, the state may require each individual to do their part by contributing to the provision of a public space. For instance, Ripstein argues that the state may tax individuals to ensure that public roads are constructed and maintained (Ripstein 2022, p. 33). Second, the state may use coercion to prevent the destruction of public spaces. To return to the roads example, the state may use coercion to prevent people from ‘privatising’ public roads—the person who parks in the middle of the road, effectively prevents others from making use, thereby limiting their freedom. Here, the state hinders the hindrance to freedom—they can use coercive power to ensure that individuals do not block public roads.

Hopefully, the reader can see how this analogy applies to vaccination. We’ll use ‘the constituency of the vulnerable’ to refer to people who cannot safely be vaccinated, have yet to be vaccinated, are immunocompromised, or for whom the vaccine was ineffective.^28^ If enough people get vaccinated and the population enjoys sufficient community protection (often referred to as ‘herd immunity’), the constituency of the vulnerable can safely make use of public spaces. If an insufficient number of people get vaccinated, however, then the constituency of the vulnerable cannot safely make use of public spaces because they face significant risk of infection and subsequent illness. If we accept that the state can use coercion to ensure the use of public spaces, then, it would seem that the state could use coercion to ensure that a large enough percentage of the population gets vaccinated so as to enjoy community protection.

At this stage, readers might insist that the analogy between other public spaces—e.g., public roads—and vaccination breaks down. After all, people can still go outside even if others decide to go unvaccinated. What’s needed to secure the analogy concerns the threat that vaccination prevents. So, to return to the public roads example, imagine that a large group of individuals insist on using heavy vehicles—e.g., tanks—that are hard on roads. Taken collectively, these individuals create potholes and other structural defects in the public roads. Such defects, in turn, make driving much riskier—motorists who don’t use heavy vehicles are far more likely to suffer damage to their automobile or even their body, in the form of an injury from a crash. In this scenario, there is a sense in which each is still free to pursue their ends by using the public roads. Yet restricting the use of heavy vehicles would protect the property and person of everyone else. And, as we have seen, damaging the person or property of others effectively amounts to reducing their freedom, for the Kantian. Moreover, the state could justifiably insist that by using the relevant vehicles, individuals damage commonly owned property. Just as the state may permissibly prevent individuals from parking park their automobile in the middle of the road on the grounds that doing so precludes the use of public roads, the state may permissibly restrict the use of vehicles that are so damaging to the road that it makes the use of the public road dangerous.

If this argument about the case of public roads works, then it seems available in the case of vaccination. Taken collectively, individuals who refuse to vaccinate their children (or themselves) make going in public dangerous for the constituency of the vulnerable. There is a sense in which the constituency of the vulnerable could still go out in public. But if the state can protect the property and person of motorists by restricting how people may make use of public spaces in the public roads case, there’s (at least) a *prima facie* case for thinking that the state can do the same when it comes to vaccination.

A final appealing feature of the right to public use argument is that it provides an explanation as to why vaccine mandates should concern *public* spaces, in particular. If individuals wish to drive their heavy vehicles on their own property and nowhere else, this poses no threat to the free use of roads. If they use public roads, however, this does endanger the free use of others. Similarly, if people were to merely remain in private spaces while unvaccinated, this would not pose a threat to public health. But if one sends one’s unvaccinated children into public spaces—such as schools or public playgrounds—then this endangers the safety of those spaces for the constituency of the vulnerable.

### Independence from vaccine-refusers

Once we recognise how the choices of vaccine-refusers can endanger the constituency of the vulnerable, we can see a second Kantian argument in defence of vaccine mandates. The constituency of the vulnerable is made dependent on the choices of vaccine refusers. This dependency, in turn, constitutes an impediment to the equal freedom of such individuals. Coercive vaccination policy would prevent this loss of freedom, thereby guaranteeing the systematic enjoyment of the right to freedom.^29^ Since, according to the Kantian, a legitimate state may permissibly use coercion to guarantee the systematic enjoyment of the right of freedom, the Kantian state may implement a vaccine mandate.

To substantiate this argument, consider (again) the following analogy on the state provision of public roads (Ripstein 2009, pp. 243–252). Imagine a world where every piece of land is privately owned. Because of property rights, each owner is entitled to completely determine how their land will be used. That includes the right to exclude other people from their land. In this world, your land is completely surrounded by other people’s lands. To go off your land, you thus need permission from your neighbours. This means you can become trapped on your own piece of land.

Now, imagine you want to meet up with a friend who lives on the other side of your neighbour. You and your friend are unable to reach each other, except by passing over your neighbour’s land. Therefore, you and your friend need your neighbour’s permission to be able to meet each other. The problem here is not that the neighbour can deny you and your friend access to their property—the real problem is that the neighbour gets to decide whether you and your friend are allowed to meet each other at all. Because the neighbour has this power, you and your friend have become subjects to your neighbour, which infringes your right to freedom as independence. Even if the neighbour would allow you to pass through, the problem stands, because you are dependent on your neighbour’s choice, just like enslaved persons are dependent on the enslaver’s consent. Your innate right to freedom as independence is infringed.

Here, there is a need for the state to protect the Universal Principle of Right. A law can be introduced dictating that everyone must have access to public roads so that no one will be dependent on other people’s choice in determining which purposes to choose and to pursue—in this case, where to go and to meet up with others. Such a law would guarantee the systematic enjoyment of the right to freedom and is therefore a justified form of state coercion.

Returning to vaccination policy, imagine, with keeping in mind the assumptions as presented earlier, that a member of the constituency of the vulnerable is surrounded by vaccine refusers. That vulnerable person will be hindered in their freedom as independence because it becomes difficult or even impossible for them to pursue their ends. For example: a vulnerable person wants to engage in public life during the epidemic, such as participating in a social activity or working with lots of people present—but without facing the risk of contracting a preventable, very harmful infectious disease. In effect, that individual faces high risk of being infected with the dangerous disease because of widespread vaccine refusal, combined with the lack of individual protection from infection because of not being individually vaccinated. These risks mean that various options and important purposes have become unacceptably costly for the vulnerable person.

Were it not for the vaccine refusers, vulnerable persons would have been able to pursue their willed goals. Because of the choice of the vaccine refusers to not get vaccinated, however, they are unable to go out in public without significant risk of getting sick. Vaccine refusers thus reduce the vulnerable persons’ ability to set and pursue their own ends, and thereby vaccine refusers make vulnerable people dependent; it is through their decision that makes that vulnerable persons can no longer effectively use their powers. Therefore, vaccine refusal can be seen as a case of destroying or injuring another’s powers (see Sect. 1), which is an objectionable form of interference with freedom: vaccine refusers violate the Universal Principle of Right. In other words, vulnerable citizens can no longer be their own master—they lack freedom as independence—and are constrained by the choice of vaccine refusers.

### Dependence on the grace of others

A third argument begins with the Kantian claim that the state may justifiably enact policies that protect individuals against becoming dependent on the will of others. Such dependence infringes the right to freedom as independence. For example, the state may justifiably raise taxes in order to provide for the poor, thereby preventing the poor from becoming completely dependent on private beneficiaries. Without such state provision, poor people would depend on the choices of others in all purposes they would try to pursue.^30^

In the context of the vaccination debate, our “dependence on the grace of others argument” begins with the observation that when there is widespread vaccine refusal, vulnerable citizens will become dependent on their beneficiaries. Not being able to safely go into public spaces precludes individuals from securing necessities. For example, in the absence of widespread vaccination against measles or polio, vulnerable people risk getting infected when they go to the grocery store or working as a cashier there. For such individuals to enjoy safety, they will require benefactors who provide them with groceries, or the means to purchase various goods. This situation is not consistent with the Kantian ideal of a system of equal freedom as independence. Ripstein (2009, p. 282) puts it: “To depend on the grace of another (…) reduces a person to the status of a thing.”

Just like the state is justified to implement policies that decrease the dependency of poor people on beneficiaries by providing for them, the state may also justifiably take steps to decrease the dependency of vulnerable people on beneficiaries. One such way to do so is by introducing vaccination policy aimed at increasing vaccination rates. In both cases, without state intervention, the former party is dependent on the assistance and grace of the latter party, making the former party’s pursuit of necessities practically impossible without assistance, thereby violating the right to freedom as independence. That is why a Kantian state is justified to implement coercive vaccination policy, to reduce this risk for dependence on others.

### Indeterminacy and bodily rights

Before moving onto the next section, a natural worry warrants discussion. Namely, coercive vaccination policy aimed at increasing vaccination rates *itself* violates the freedom of vaccine refusers. After all, threatening people in order to get them to behave in a way that they otherwise would not certainly seems to be a paradigmatic case of usurping their freedom.

If vaccine mandates constitute a hindrance of a hindrance to freedom, however, then they would not be an objectionable instance of coercion. And we have contended that such mandates *can* constitute a hindrance of a hindrance to freedom—they can correct the hindrance vaccine refusers inflict on vulnerable persons, which makes it a restoration of the original right to freedom as independence. This restorative character renders the policy a rightful form of coercion. Furthermore, it is enough for Kantians that the law ensures the preconditions of freedom, not that all individuals would necessarily like the introduction of that law.^31^ We contend that introducing coercive vaccination policy that increases vaccination rates is such a law. The coercive character of vaccine mandates, then, hardly suffices to undercut the Kantian justification we have offered.

At this juncture, let us assume that the arguments so far are sound: vaccine mandates can protect the freedom of some, and they do not objectionably constrain the freedom of those who do not wish to be vaccinated. Still, there seems to be an important disanalogy between the government mandating vaccines and the government ensuring the availability of public roads or ensuring individuals can avoid freedom-undermining dependency. Namely, vaccine mandates—unlike these other examples of justified government coercion—appear to infringe upon rights individuals have over their *bodies*. And in debates about mandates, vaccine refusers often argue against coercive vaccination policy by referring to their bodily rights—claiming their rights over their bodies include a right to not get vaccinated. Indeed, opponents of mandates have invoked slogans used in defence of reproductive rights—e.g., “My body my choice”—when articulating their objections.^32^

In response, it is beyond question that bodily rights are important for Kantians. And, so, it might be tempting to think that given their commitments to bodily rights, Kantians would oppose vaccine mandates. Yet the rights to one’s body are indeterminate. This indeterminacy combined with the proper role of the state in resolving such indeterminacy allows the Kantian to plausibly respond to the objection from bodily rights.

As we have noted above, Kantians contend there will always be disagreements about the limits and extent of rights. The source of this indeterminacy problem is not empirical or epistemic, but normative and formal: there are not straightforward a priori answers to such disputes to be found in the state of nature. Importantly for present purposes, however, this point extends to rights over one’s own body. Private citizens cannot, in a state of nature, determine among each other what the boundaries of bodily rights must be because this will be a situation where arbitrary individual force prevails. Furthermore, as discussed before, no unilateral will (the will of a private citizen, including their ideas on the extent of their bodily rights) can make laws and impose obligations on others. That is why a (judiciary branch of) government—the omnilateral will—is needed to apply law to particular cases, solve the indeterminacy problem found in a state of nature, and to be a publicly accepted authority for resolving legal disputes (Ripstein 2009, pp. 168–176).

To illustrate this indeterminacy problem of bodily rights in the context of the vaccination debate, it will help to compare bodily rights with property rights. In a state of nature, it is indeterminate how long intellectual property rights last and when something becomes public domain, or how deep under the earth and into the sky your property rights to your plot of land extend. Similarly, in a state of nature it is indeterminate how far one’s bodily rights extend—at what point your rights over your body begin and someone else’s end. For instance, in the context of self-defence, it is indeterminate from which distance another person can be seen as a threat to your body, and what bodily acts should be counted as self-defence or aggressive action (Hasan 2020). Presumably, merely looking at someone does not constitute aggressive action that licences self-defence. At the same time, it would be implausible that one’s rights to self-defence only arise when someone else’s fist is inches away from one’s face. More generally, it is indeterminate which acts I perform with my body in the close presence of other people are rightful and which are not. Public law is therefore needed to determine these boundaries of bodily rights. As Pallikkathayil (2017, p. 36) puts it: “We are thoroughly political beings; even our rights to our own bodies are politically constructed.” Coercive vaccination policy is also subject to this indeterminacy. At what point does exposing others to risk of infection constitute a violation of their rights?^33^ The first thing to note, then, is that the Kantian apparatus is responsive to this feature of our moral disagreements about coercive vaccination policy; our disagreements are, in part, disagreements about the extent of the rights over one’s body.

More importantly, however, this indeterminacy and the Kantian outlook allow a defence against the objections from bodily rights. If bodily rights are indeterminate, and if the state (and only the state) should resolve this determinacy, then on the Kantian framework the state is necessary and uniquely justified in determining the boundaries over where one person’s rights end and another person’s rights begin. A vaccine mandate responds to this indeterminacy by deciding that the bodily rights of vulnerable citizens (the rights to not get infected and to remain free and independent) constrain the choices of vaccine refusers with regard to their own bodies.

To be clear, the indeterminacy and bodily rights argument does not, on its own, justify mandatory vaccination policies. But it does explain why one of the most prominent and ostensibly powerful lines of objection to such policies is not as compelling as its proponents claim. Where my rights over my body stop and the rights of others over their bodies begin is indeterminate, generally. Whether vaccine mandates are morally justifiable, then, cannot be decided simply by appealing to bodily rights. Given the positive arguments for vaccine mandates that draw on Kantian political philosophy, however, we have seen that the Kantian can make a compelling case in defence of vaccine mandates. Accordingly, we have shown how the Kantian framework can both be used to advocate for vaccine mandates and defend against the most powerful objection to them.

## Comparison with the harm principle

Above, we argued that the Kantian state is justified to enact coercive policies to guarantee the systematic enjoyment of that freedom as independence. In the context of vaccination ethics, we argued that according to Kantian political philosophy, the state can justifiably introduce coercive vaccination policy aimed at increasing vaccination rates. To illustrate the distinctiveness of this approach, it is worth comparing our arguments with a popular strand of argument that appears in public health ethics generally—if only to be dispensed with^34^—and the vaccination ethics debate specifically: arguments based on the liberal conception of freedom as non-interference and the harm principle.

Mill (1859/2015, p. 13) originally articulated what has come to be called ‘the harm principle’ as follows: “the only purpose for which power can be rightfully exercised over any member of a civilized community, against his will, is to prevent harm to others.” The harm principle thus entails that a government can only interfere with individual freedom to prevent harm to third parties. So, if the results of an individual’s act are harmful to other people, the government can interfere with that act. This reasoning seems, prima facie, also applicable to vaccination. Indeed, many have advanced arguments that invoke the harm principle to justify vaccine mandates (Bambery et al. 2013; Brennan 2018; Dawson 2007, pp. 166–171; Dawson 2011, pp. 144–145; Flanigan 2014; Giubilini 2019, p. 33; Gostin 2003, pp. 1145–1148; Holland 2015, pp. 362–363; Luyten et al. 2011, pp. 282–283; Navin 2015, pp. 173–176; Navin and Atwell 2019; Savulescu 2021; Vermeersch 1999; Verweij 2011, pp. 108–113). The general idea is as follows: vaccination prevents infection with potentially dangerous infectious diseases, so when many citizens are vaccinated, transmission will be significantly lowered. If many citizens choose to refuse vaccination for themselves (or their children), the risk for others to get infected will be increased. Because that situation is harmful for those third parties, especially for vulnerable people, the state is justified to introduce a vaccine mandate to prevent that harm to those other people. Vaccine mandates are thus a way of preventing individuals (vaccine refuses) from imposing an unjust risk of harm on third parties, so they do not run afoul of the harmful principle.

An upside for arguments based on the harm principle is that they are not paternalistic; the reason for government intervention has nothing to do with potential harm caused by vaccine refusal inflicted to the vaccine refusers themselves, but rather with the harm inflicted by the vaccine refusers to third parties (Dawson 2011, p. 145). Therefore, the arguments do not fall prey to the same criticism that paternalistic arguments do, and they fit better with core tenets of liberal political morality. Additionally, such arguments feel intuitively right and familiar, as they can be seen in the light of the influential bioethical principle of non-maleficence (Beauchamp and Childress 2019; Chap. 5).

### Problems for the harm principle

An important problem with applying the harm principle in the context of vaccine mandates is that it is questionable if one individual refusing vaccination actually poses a sufficiently significant risk of harm to others—especially in contexts where the population enjoys herd immunity (Giubilini 2019; Bernstein 2021). At least two considerations are relevant in this context.

The first consideration is that the harm imposed by vaccine refusal on third parties is most likely to be indirect. Rather than vaccine refuser A directly infecting vulnerable person X (direct harm imposition), it is more likely that vaccine refusers A, B, and C together contribute to increased disease circulation, which leads to a (multistep) spread of the disease to vulnerable persons X, Y, and Z (indirect harm imposition). Some also include the indirect harm widespread vaccine refusal will impose on the health care system in general, as vaccine refusers will likely have higher consumption of health resources, thereby indirectly harming other people who (also) need health care treatment. While direct harm imposition can clearly count under Mill’s harm principle, it is unclear if such indirect kinds of harm imposition also can, given the variety of interpretations of the harm principle (Brink 2022; Turner 2014; White 2024).

The second and more important consideration is that in contexts where herd immunity exists, the risk of harm imposed individually by a vaccine refuser on third parties is so little that it is negligible: one individual refusing vaccination has a negligible effect on herd immunity and—in contexts where herd immunity exists—on the chance of others to get infected (Dawson 2007, pp. 169–170; Giubilini 2019, p. 33; Bernstein 2021). A vaccine refuser may thus plausibly claim that if their act of vaccine refusal poses so little risk of harm to others that it is negligible, the state is not allowed to coerce them to get vaccinated based on the harm principle.

Some proponents of harm prevention arguments have tried to work around this objection. Flanigan (2014), for example, claims that the state is nonetheless permitted to compel individuals to get vaccinated, because while going unvaccinated imposes a very small risk on others, it imposes a *deadly* risk. She compares vaccine refusal with firing a rifle in the air on a busy holiday. Firing a rifle in the air imposes a very small risk on others, but a relevant one, because it is a deadly risk. Therefore, she argues, we have no right to fire a rifle in the air, and thus we also have no right to refuse vaccination.

However, Flanigan’s argument is questionable for two reasons. First, lots of other behaviours, such as people driving cars, also impose small but deadly risks on others (perhaps even bigger risks than vaccine refusal), but these behaviours are not believed to be morally wrong nor to be coercively prohibitable. So why should vaccine refusal be different? Second, the comparison between firing a rifle in the air and vaccine refusal does not hold, because vaccine refusal only imposes a deadly risk on others if many others also refuse vaccination: it is primarily a contribution to a risk of collective harm (Bernstein 2017, pp. 793–794; Giubilini 2020, p. 448).

Acknowledging the problems with Flanigan’s argument, Brennan (2018) notes that vaccine refusal is a collective action problem. He claims that there is a coercively enforceable moral obligation to not participate in collectively harmful activities (the “clean hands principle”), and that refusing vaccination is such a collectively harmful activity that exposes others to an unacceptable risk. Therefore, the state would be justified to introduce a vaccine mandate based on preventing-harm-to-others reasoning. However, there is still an overgeneralisation worry here. Other activities, such as driving polluting cars, also count as participating in a collective harmful activity (in this example, causing climate change), but are not generally believed to be coercively prohibitable because of the negligible contribution of one individual, especially not in the libertarian framework Brennan accepts (Bernstein 2017, p. 795; Giubilini 2020, p. 449). As Giubilini puts it: “whether my hands are clean or dirty does not make a difference to whether herd immunity is realized” (Giubilini 2019, p. 100). So, because of the negligible consequences of an individual act of vaccine refusal on third parties, proponents of vaccine mandates need something else than the harm principle to be able to justify vaccine mandates.^35^

### The Kantian alternative?

Now, how does Kantian freedom compare to arguments that invoke the harm principle? A first interesting advantage of the Kantian argument is that the Kantian argument does not focus on (harmful) outcomes that result from an individual act, but rather on the act itself. Ripstein (2006, p. 218) puts it: “The harm principle is forced to focus on how my deed affects you, rather than on what it is that I do to you.”

An illustrative example is harmless trespassing on someone else’s property. This trespassing will not do any harm to the owner of the property but is still wrong: not because of the harmful outcomes of the trespassing, but because of the trespassing itself, which infringes other people’s freedom, and thereby violates the Universal Principle of Right. So, just like harmless trespassing is still wrong—even in the absence of harm—vaccine refusal with negligible harmful consequences is also still wrong, because they both infringe the system of equal freedom for all, violating the Universal Principle of Right.^36^

Furthermore, the Kantian approach differs importantly from the Millian approach because it is not focussed on harm or wellbeing but on freedom. So, an upside for a Kantian justification for coercive vaccination policies is that it does not need to prove that vaccine refusers really impose significant harm on other people to justify vaccine mandates. It only needs to show that vaccine refusal, taken collectively, compromises or conditions other people’s freedom.

The fact that the Kantian arguments appeals to freedom has an additional appealing feature that we wish to highlight. Often, debates about vaccine mandates or other public health measures portray the issue as a conflict between two values: freedom and well-being, or something along those lines. The framing of the issue, then, turns on how we should weigh the value of freedom against rival values. In this view, public health measures are justifiable because they realise some value other than freedom, and the significance of realising this other value outweighs or overrides the value of freedom. Yet it is not always clear why these other values should outweigh or override freedom—especially when opponents of a given policy seem to place greater weight on freedom. This is a familiar problem for any pluralist approach to ethics; people disagree about the relevant weights of competing values, and so insisting that these other values should give way to freedom risks begging the question against opponents of such interventions.

The appealing feature of the Kantian view for proponents of public health interventions is that it can justify coercion by appealing to one of the central values that opponents of such interventions invoke. Consider those vulnerable citizens during the COVID-19 pandemic who became dependent on the will of others for assistance (delivering groceries, etc.) because it was too unsafe for them to go outside. Intuitively, they surely lost an important part of what it entails to be free. Now, it is an open question about whether appealing to freedom or other considerations will be most likely to convince opponents of public health measures, and we lack the space to make—let alone assess—the case for the effectiveness of appealing to different sorts of values to justify various policies, especially if we consider different cultural and political contexts where appeals to freedom have a more or less central place. Nonetheless, a potentially appealing feature of the Kantian approach is how it justifies coercive policies in terms of freedom rather than simply by insisting that freedom should be subordinate to other values.

## Conclusion

In this article, we have discussed Kantian political philosophy as a theoretical resource for evaluating the moral justification of coercive public health interventions. To make the general promise of this approach more concrete, we illustrated how Kantian political philosophy can be used to justify coercive vaccination policies aimed at increasing vaccination rates. We also highlighted a few advantages of the Kantian approach compared to Mill’s harm principle as well as pluralist approaches that frame public health interventions as trade-offs between, say, liberty and overall well-being.

Our arguments hardly demonstrate that the Kantian approach to coercion in public health ethics is, all-things-considered, superior to its rivals. And we anticipate that many will find this framework unconvincing or problematic for reasons that have been advanced in other contexts. For instance, some might object to the importance of securing freedom as independence on the grounds that it fetishises independence, or they might think that the Kantian approach to public health ethics is too conservative because its emphasis on securing freedom does not justify policies that are more straightforwardly aimed at improving well-being, nor does it straightforwardly address inequalities that do not undermine equal freedom. Conversely, more classical liberal and libertarian theorists endorse a far thinner understanding of freedom than Kantians, and thus they would likely object to our contention that freedom requires various coercive public health policies. What we hope our arguments do demonstrate, however, is that Kantian approaches to political philosophy should be taken more seriously in public health ethics than they have been.

## Footnotes

1 See Faden et al. (2022).

2 There have been some discussions of Kantian ethics and political philosophy with an eye to healthcare entitlements—see, e.g., Hughes (2020)—but to our knowledge, there has been minimal reference to Kantian political philosophy when discussing the role of government in protecting population health.

3 See, e.g., Beauchamp and Childress (2019). For the critique that these are not, in fact, based on a Kantian understanding of autonomy, see esp. Reis-Dennis (2020).

4 See, e.g., De Miguel Beriain (2021), Pence (2021).

5 See Mill (1859/2015), and e.g., Faden et al. (2022, 3.2.2).

6 See Nuffield Council on Bioethics (2007, 3.37).

7 See, e.g., Holland (2015; Chap. 1).

8 See, e.g., Baylis et al. (2008).

9 See, e.g., Dawson and Jennings (2012). Others have explored broadly ‘republican’ approaches to public health ethics—for an overview, see Faden et al. (2022). These approaches do not focus on coercion, per se, and they tend to be framed in more consequentialist terms. See, e.g., Pettit (1997) for this framing.

10 “Freedom (independence from being constrained by another’s choice), insofar as it can coexist with the freedom of every other in accordance with a universal law, is the only original right belonging to every man by virtue of his humanity.” (Kant 1797/1996b, 6:237).

11 Sometimes, other definitions of Kantian freedom are given, such as “freedom as sovereignty” (Ripstein 2006, 2009, p. 35), “external freedom” (Ripstein 2004, p. 8), and “freedom as non-domination” (Ripstein 2009, p. 42). Freedom as independence is, however, the most frequently used definition, and where other terms are used, they are used synonymously, pointing to the idea that freedom means that everybody has the right to be independent from the choices of others under universal law. For a different account of how Kant’s innate right should be interpreted, see for example Flikschuh (2022).

12 Importantly, “constraint” does not include the mere frustration of the wishes of others (Ripstein 2009, p. 19).

13 This does not mean per se that their goals must be fulfilled, because that fulfilment depends on the means one has (their own bodily and intellectual means or other, external, means).

14 See, e.g., Ripstein (2009), p. 14.

15 Compare this with Kant’s Formula of Humanity, formulated in his discussion of his practical philosophy as follows: “So act that you use humanity, whether in your own person or in the person of any other, always at the same time as an end, never merely as a means.” (Kant 1785/1996a, 4:429). See Ripstein (2009, pp. 355–388) for further comparisons between Kant’s legal and political thought (which is the main interest of this paper) and Kant’s practical philosophy (including his famous Categorical Imperative).

16 This Universal Principle of Right should be distinguished from Kant’s Formula of Universal Law. The latter, “act only in accordance with that maxim through which you can at the same time will that it become a universal law” (Kant 1785/1996a, 4:421), is one of Kant’s categorical imperatives as put forward in his moral philosophy.

17 Kantians claim that from the innate right to freedom in your own person, there follows a right to be free to use other means for pursuing one’s own chosen ends, without being made subject to another person’s choice. Most fundamentally, we need external means to survive—in the form of food and water. But also, for various kinds of long-term planning, we need to be able to use—and count on using—means to pursue our ends. These entitlements fall under the category of private right. An important private right is property right: the right to things (Ripstein 2009, pp. 19–20).

18 For more on the ‘omnilateral will’, see Kant (1797/1996b, 6:263), Pallikkathayil (forthcoming), or Ripstein (2009; Chap. 7).

19 In this paper, we are not able to extensively discuss these three requirements; however, we will return to the role of the state in providing determinacy later on. For a detailed discussion of the requirements that ensure a rightful condition, see Ripstein (2009; Chap. 6).

20 Kantians argue that private rights, such as property rights, can only be “conclusive” (decisive, complete; as opposed to “provisional”) when these rights are made, applied, and enforced by the state (see Kant 1797/1996b, 6:255; Ripstein 2009; Chap. 6). In other words, they can only be conclusive when there is a rightful condition.

21 Some Kantians disagree that the innate right to freedom as independence grounds a system of public policy and state coercion. One reason for that would be that the distinction between actions that subject some people’s wills to other people’s choices and actions that merely effect the contexts in which people act, and therefore the distinction between actions that the state can forbid and actions that the state should allow, would be unclear (see, e.g., Sangiovanni 2012; Valentini 2012). We leave this debate aside, as we follow Ripstein’s interpretation. See Ripstein (2012) for his response to Sangiovanni’s and Valentini’s critiques.

22 For an argument to the contrary, based on a different interpretation of Kant, see Stemplowska (2022).

23 For a definition of vaccine mandates, see esp. Atwell and Navin (2019).

24 For an account of the moral significance of herd immunity for vulnerable people, see esp. Bernstein and Navin (2023).

25 See esp. Kant (1797/1996b, 6:246–247).

26 For more on the indeterminacy problem, see Sect. 2.4.

27 One might question why the state should be the default decider what the boundaries to various rights should be. We might ask, for instance, why this could not be decided entirely through convention, or through some informal decision procedure (e.g., a lottery) that resolves such disputes. The Kantian reply is that the state is preferable because it can enjoy recognition as the appropriate authority for resolving such disputes, recognition that can be denied to private persons or informal procedures. Moreover, the state acts as the collective body with which its citizens rule themselves, embodying the unilateral will to ensure the rightful condition—and it can be recognized as such. See, e.g., Ripstein (2022). We thank an anonymous reviewer who pressed us to explain this.

28 For this phrase, see Bernstein and Navin (2023).

29 For a distinct argument that contends that *lockdowns* preserve freedoms—albeit without a Kantian understanding of freedom—see Oberman (2022).

30 In this, we follow Ripstein’s interpretation. He claims: “Poverty, as Kant conceives it, is systematic: a person cannot use his or her own body, or even so much as occupy space, without the permission of another. The problem is not that some particular purpose depends on the choices of others, but that the pursuit of any purpose does. If all purposiveness depends on the grace of others, the dependent person is in the juridical position of a slave or serf” (Ripstein 2009, p. 281). Other Kantians have questioned Ripstein’s interpretation. Hasan (2018), for example, agrees the Kantian account justifies state-mandated economic redistribution, but claims this justification is not rooted in the rich’s direct control over the choice-making capacities of the poor, but in a fundamental problem of structural disempowerment. Davies (2020), on the other hand, doubts there are Kantian grounds for state provision of welfare at all, and thinks Ripstein misrepresents the relation between the rich and the poor, while Hasan deviates too much from Kantian freedom as independence. We cannot go into more detail on this discussion here, and follow Ripstein’s interpretation. For more on this discussion, see for example Allais (2015); Penner (2010); Weinrib (2003).

31 See Gregory (2023) or Varden (2010).

32 For in-depth discussion of this comparison, see Rulli and Campbell 2022.

33 For a similar formulation, albeit in a much more libertarian refrain, see Brennan (2018).

34 See Faden et al. (2022); Gostin and Gostin (2009).

35 For an additional critique of the use of the harm principle, one that focuses on how infectious disease control can protect institutions or other more diffuse negative outcomes than individual harm, see White 2024. We think that the emphasis on the role of government institutions in securing equal freedom provides a promising route for the Kantian to accommodate White’s point—although we lack the room to explore this issue at adequate length.

36 A critic might claim that, besides the absence of harm, one individual refusing vaccination does also not significantly infringe another individual’s freedom—in other words, that one act of vaccination has negligible effects on freedom. However, as stated earlier, according to the Kantian people can still be compelled to contribute to the system of equal freedom, without relying on the consequences those individual contributions will actually bring (Ripstein 2022, p. 25), rendering that critic’s claim irrelevant.
